# Inhibition of mTOR Prevents ROS Production Initiated by Ethidium Bromide-Induced Mitochondrial DNA Depletion

**DOI:** 10.3389/fendo.2014.00122

**Published:** 2014-07-24

**Authors:** Timothy Nacarelli, Ashley Azar, Christian Sell

**Affiliations:** ^1^Department of Pathology, Drexel University College of Medicine, Philadelphia, PA, USA

**Keywords:** mitochondria, ROS, mTOR, rapamycin, senescence, aging, ethidium bromide, p38

## Abstract

The regulation of mitochondrial mass and DNA content involves a complex interaction between mitochondrial DNA replication machinery, functional components of the electron transport chain, selective clearance of mitochondria, and nuclear gene expression. In order to gain insight into cellular responses to mitochondrial stress, we treated human diploid fibroblasts with ethidium bromide at concentrations that induced loss of mitochondrial DNA over a period of 7 days. The decrease in mitochondrial DNA was accompanied by a reduction in steady state levels of the mitochondrial DNA binding protein, TFAM, a reduction in several electron transport chain protein levels, increased mitochondrial and total cellular ROS, and activation of p38 MAPK. However, there was an increase in mitochondrial mass and voltage dependent anion channel levels. In addition, mechanistic target of rapamycin (mTOR) activity, as judged by p70S6K targets, was decreased while steady state levels of p62/SQSTM1 and Parkin were increased. Treatment of cells with rapamycin created a situation in which cells were better able to adapt to the mitochondrial dysfunction, resulting in decreased ROS and increased cell viability but did not prevent the reduction in mitochondrial DNA. These effects may be due to a more efficient flux through the electron transport chain, increased autophagy, or enhanced AKT signaling, coupled with a reduced growth rate. Together, the results suggest that mTOR activity is affected by mitochondrial stress, which may be part of the retrograde signal system required for normal mitochondrial homeostasis.

## Introduction

Mitochondrial dysfunction is associated with a variety of clinical disorders including Parkinson’s disease, Friedreich ataxia, neurodegeneration, aging, and multiple clinical myopathies, (1–3). Integrity of the mitochondrial genome is critical to normal function as demonstrated by pathological settings in which mitochondrial DNA is lost leading to a variety of clinical manifestations, which include myopathic, encephalomyopathic, hepatocerebral, and neurogastrointestinal symptomatology (4). A direct link between age-associated loss of function and mitochondrial DNA integrity is supported by the fact that transgenic mice harboring a proof-reading deficient DNA polymerase gamma exhibit cardiomyopathy and premature aging (5).

The circular mitochondrial genome is approximately 16,569 base pairs in humans (6), and ranges from 1000 to 8000 copies per cell (7). The entire mitochondrial genome is dedicated to energy production, coding for 13 electron transport chain (ETC) protein subunits 2 rRNA and 22 tRNA genes (8–10). Replication of mtDNA is on-going throughout the cell cycle and is independent of mitosis (11–14). Prolonged exposure to concentrations of ethidium bromide that do not impair cell proliferation leads to the specific loss of mitochondrial DNA. This effect is caused by disruption of the tertiary structure of the closed circular mitochondrial genome following intercalation of ethidium bromide (15) leading to inhibition of both transcription and replication in the mitochondria without while sparing both nuclear DNA synthesis and transcription of nuclear encoded mRNAs (16–19). Nanogram to microgram concentrations of ethidium bromide per milliliter of culture medium induces loss of mitochondrial DNA and complete loss of mitochondrial DNA occurs after 5–6 weeks of exposure (19–21), and this approach has facilitated the study of mitochondrial mutations through introduction of specific mutant mitochondrial genomes (19, 22). In addition, the response to mitochondrial DNA depletion can be examined using this approach. Studies in cells that have lost mitochondrial DNA over a period of weeks in culture report increased mRNA levels of genes involved in hypoxic response, mitochondrial ribosomal proteins, solute transport, and glycolytic pathways (23), while mitochondrial transcripts are increased in cells allowed to recover from mtDNA depletion (21, 24).

In this study, we have examined the response of human diploid fibroblasts (HDF) to a series of concentrations of ethidium bromide in order to gain a better understanding of the cellular responses to mitochondrial stress.

## Materials and Methods

### Cell culture

Normal human fibroblast cells (WI-38) were cultured in Minimum Essential Medium Eagle (MEM) supplemented with 1% l-glutamine, MEM amino acids and vitamins, and 10% fetal bovine serum according to standard culture protocol (25). Cells were maintained by trypsinization and reseeding at a cell density of 1 × 10^4^/cm^2^ every 7–10 days. All culture reagents were from Cellgro (Manassas, VA, USA).

Cultures were exposed to ethidium bromide in full growth medium supplemented with 50 μg/ml uridine and 1 mM sodium pyruvate containing the indicated concentrations of ethidium bromide indicated in each experiment (26). For the ethidium bromide treatment, cells were seeded at a standard density (1 × 10^4^/cm^2^) according to a protocol established for the cultivation of human mesenchymal cell cultures (27). Ethidium bromide containing medium 24 h post seeded to ensure that the drug did not alter cell attachment. Cultures were exposed to ethidium bromide containing medium for 7 days with one change of medium. Cell viability was assessed after ethidium bromide treatment using trypan blue exclusion. Cells were trypsinized, re-suspended in growth media, and diluted 1:1 using 0.4% trypan blue solution. This mixture was incubated at room temperature for 1–2 min then loaded onto a hemocytometer for visual assessment of dye uptake. For cell imaging, cells were infected with a mitochondrial GFP-containing virus from the University of Pittsburgh vector core. Cells were exposed to virus in the presence of 10 μg/ml polybrene and utilized as a mixed population without selection. After treatment with ethidium bromide and/or rapamycin, cells were imaged on an advanced microscopy group EVOS inverted fluorescent microscope at a constant intensity of 20% using a 60× objective.

### Analysis of mitochondrial DNA copy number

Mitochondrial DNA was extracted using phenol–chloroform extraction based upon published analysis of methods for the optimal extraction of mitochondrial DNA (28). Total DNA was extracted using phenol/chloroform extraction using the phase-lock gel system (5Prime Inc., Gaithersburg, MD, USA) followed by ethanol precipitation and a second phenol/chloroform extraction. Total DNA was quantified using a nanodrop spectrophotometer. Primers used to amplify mitochondrial and nuclear DNA targeted the mitochondrial leucine tRNA transcript and the nuclear beta-2 microglobulin gene locus. Primer sets were as follows: for leucine tRNA, 5′-CACCCAAGAACAGGGTTTGT-3′ and 5′-TGGCCATGGGTATGTTGTTA-3′: for beta-2 microglobulin, 5′-TCTCTGCTCCCCACCTCTAAGT-3′ and 5′-TGCTGTCTCGATGTTTGATGTATCT-3′. Quantitative real-time PCR was performed using the Verso Sybr 1 step qPCR kit (Thermo Scientific, Waltham, MA, USA) in a Stratagene Mx3000P thermocycler.

### Protein isolation and immunoblot analysis

Total protein extracts were prepared through extraction with RIPA buffer and proteins quantified using a bicinchoninic acid assay (Pierce Biotechnology, Rockford, IL, USA). For Western blot analyses, 30 μg of protein extracts were run on SDS-PAGE and transferred onto Immobilon P PVDF (Millipore, Billerica, MA, USA) or nitrocellulose (Bio-Rad, Hercules, CA, USA) membranes. Blots were incubated with antibodies specific for p53 (Ab-6; EMD Millipore, San Diego, CA, USA), phospho(S473)-AKT, AKT phospho(S235/236)-ribosomal protein S6, ribosomal protein S6 (Cell Signaling, Danvers, MA, USA), p62 (Enzo Biologicals, Plymouth Meeting, PA, USA), MitoProfile Total OXPHOS Cocktail (complex I-NDUFB8 subunit, CII-SDHB subunit, CIII-UQCRC2 subunit, and CIV-mitochondrial COX1 subunit) (Abcam, Cambridge, MA, USA), beta-actin (Sigma, St Louis, MO), Parkin (H-300, Santa Cruz Biotechnologies, Santa Cruz, CA, USA), TFAM (Santa Cruz Biotechnologies, Santa Cruz, CA, USA), and voltage dependent anion channel (VDAC) (4866; Cell Signaling, Beverly, MA, USA) according to manufacturer’s instructions.

### Mitochondrial potential, mitochondrial function, ROS production, and mitochondrial mass evaluation

For mitochondrial potential studies, cells were incubated with 5 μg/ml JC-1 (Molecular Probes, Carlsbad, CA, USA) at 37°C in 5% CO_2_ for 30 min, harvested in 2.5% trypsin–EDTA, re-suspended in 200 μl of complete growth medium, and analyzed immediately with a Guava Easy-Cyte Mini using the Guava Express Plus program (Millipore, Billerica, MA, USA). Live-cell imaging was performed using an EVOS FL microscope (AMG, Bothell, WA, USA) immediately following the 30-min incubation period. For mitochondrial mass evaluation, cells were incubated for 30 min in 100 nM MitoTracker Green FM (Molecular Probes, Carlsbad, CA, USA) at 37°C, harvested in 2.5% trypsin–EDTA, and re-suspended in 200 μl complete growth medium. Cells were analyzed immediately with a Guava Easy-Cyte Mini using the Guava Express Plus program. Mitochondrial ROS was measured by incubating cells with MitoSox superoxide anion indicator (Molecular Probes, Carlsbad, CA, USA) at 5 μM for 10 min in growth medium at 37°C in 5% CO_2_ for 10 min. Cells were trypsinized as above and analyzed using a Guava Easy-Cyte Mini using the Guava Express Plus program. Total cellular ROS was assessed using 2,7-dichlorodihydrofluorescein diacetate (DCFH-DA). Cells were seeded in triplicate wells and following a 24–48-h attachment period, cells were trypsinized, washed with phosphate buffered saline, and re-suspended in PBS containing 10 μM DCFH-DA. Following a 30-min incubation, cells were collected by centrifugation, re-suspended in PBS, and analyzed by flow cytometry using a Guava Easy-Cyte Mini flow cytometer.

### Statistical analysis

Unless otherwise stated in figure legend, all results are expressed as average plus or minus standard deviation of three independent samples. Significance was assessed with the unpaired, two tailed Student’s *t*-test.

## Results

**Figure 1:**
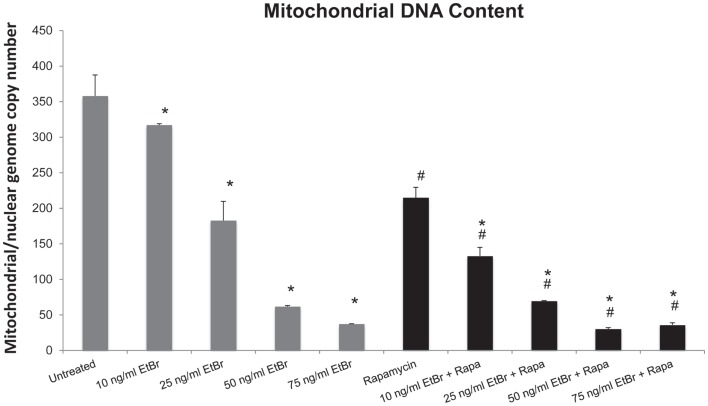
**Ethidium bromide exposure leads to a loss of mitochondrial DNA**. Human fibroblast cell cultures grown in the presence or absence of 1 nM rapamycin were exposed to increasing doses of ethidium bromide for a period of 7 days. At this time, the relative level of mitochondrial DNA was assessed using real-time PCR analysis as described in Section “Materials and Methods.” Gray bars indicate control cultures and black bars indicate rapamycin treated cultures. The concentration of ethidium bromide is indicated beneath each bar. Differences between cultures exposed to ethidium bromide and unexposed cultures, which are significant at *P* < 0.05 are indicated by an asterisk and differences between control and rapamycin treated cultures under identical conditions are indicated by a pound sign. The graph contains a representative experiment of a minimum of three independent replicates with similar results and significance.

Human diploid fibroblasts were exposed to ethidium bromide at concentrations ranging from 10 to 100 ng/ml for 7 days. Under these conditions, we found a dose-dependent decline in the mitochondrial DNA content (Figure 1). We have previously found that long-term exposure to nanomolar concentrations of rapamycin increases replicative lifespan of HDF, reduces the percent of cells with depolarized mitochondria, and increases the expression of genes associated with mitochondrial biogenesis (25, 29). We utilized this paradigm to examine the response of rapamycin treated cells to a direct mitochondrial insult such as ethidium bromide exposure. When HDF were grown in the presence of 1 nM rapamycin and exposed to ethidium bromide, we found a similar reduction in mitochondrial DNA content, although the initial copy number was lower than control cultures (Figure 1).

**Figure 2:**
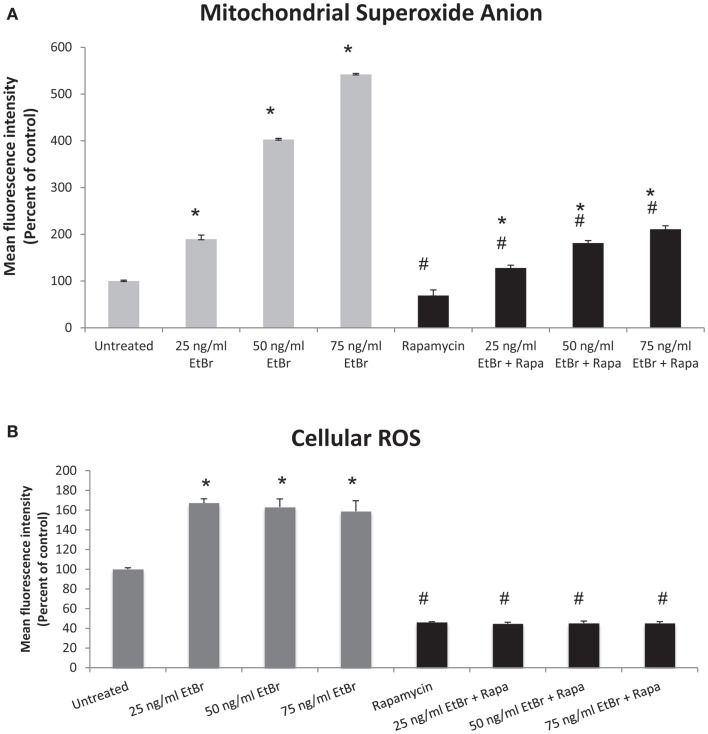
**Mitochondrial and total cellular ROS production**. Human fibroblast cell cultures grown in the presence or absence of 1 nM rapamycin were exposed to increasing doses of ethidium bromide for a period of 7 days. At this time, mitochondrial ROS and cellular ROS were measured as described in Section “Materials and Methods.” **(A)** Mitochondrial ROS production is presented. Gray bars indicate control cultures and black bars indicate rapamycin treated cultures. **(B)** Relative levels of total cellular ROS are presented. The concentration of ethidium bromide is indicated beneath each bar. Differences between cultures exposed to ethidium bromide and unexposed cultures, which are significant at *P* < 0.05 are indicated by an asterisk and differences between control and rapamycin treated cultures under identical conditions are indicated by a pound sign. The graph contains a representative experiment of a minimum of three independent replicates with similar results and significance.

**Figure 3:**
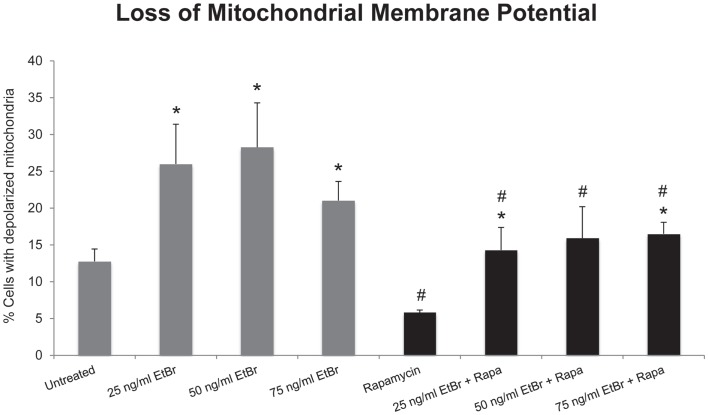
**Loss of mitochondrial membrane potential following exposure to ethidium bromide**. Human fibroblast cell cultures grown in the presence or absence of 1 nM rapamycin were exposed to increasing doses of ethidium bromide for a period of 7 days. At this time, mitochondrial membrane potential was assessed as described in Section “Materials and Methods.” Gray bars indicate control cultures and black bars indicate rapamycin treated cultures. The concentration of ethidium bromide is indicated beneath each bar. Differences between cultures exposed to ethidium bromide and unexposed cultures, which are significant at *P* < 0.05 are indicated by an asterisk and differences between control and rapamycin treated cultures under identical conditions are indicated by a pound sign. The graph contains a representative experiment of a minimum of two independent replicates with similar results and significance.

**Figure 4:**
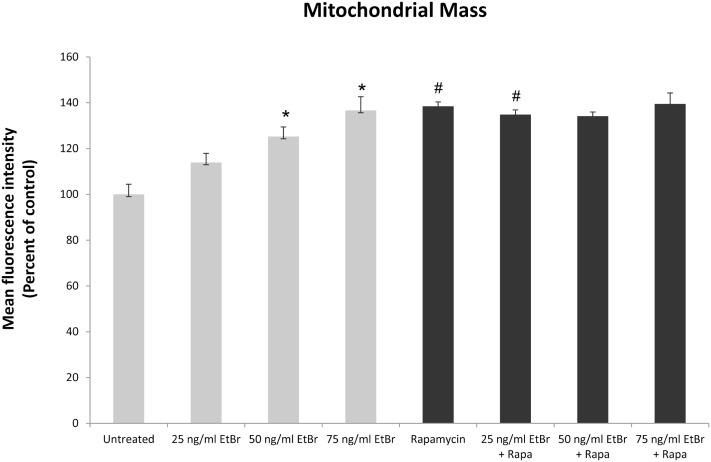
**Mitochondrial mass following exposure to ethidium bromide**. Human fibroblast cell cultures grown in the presence or absence of 1 nM rapamycin were exposed to increasing doses of ethidium bromide for a period of 7 days. At this time, mitochondrial mass was assessed using MitoTracker green as described in Section “Materials and Methods.” Gray bars indicate control cultures and black bars indicate rapamycin treated cultures. The concentration of ethidium bromide is indicated beneath each bar. Differences between cultures exposed to ethidium bromide and unexposed cultures, which are significant at *P* < 0.05 are indicated by an asterisk and differences between control and rapamycin treated cultures under identical conditions are indicated by a pound sign. The graph contains a representative experiment of a minimum of two independent replicates with similar results and significance.

We examined several aspects of mitochondria following ethidium bromide exposure. Both mitochondrial-specific and total cellular ROS were increased in cells that were exposed to ethidium bromide (Figure 2). Pretreatment of HDF with rapamycin decreased both the baseline levels of cellular ROS and significantly reduced the effect of ethidium bromide on the production of ROS (Figure 2). Similarly, the baseline levels of mitochondrial ROS levels and the mitochondrial ROS induced by ethidium bromide were significantly reduced by rapamycin pretreatment (Figure 2). The percent of cells with depolarized mitochondria was increased following ethidium bromide treatment and this percentage was reduced by pretreatment with rapamycin (Figure 3). Despite the reduction in mitochondrial DNA, mitochondrial mass was increased following ethidium bromide treatment in a dose-dependent manner, while HDF grown in the presence of rapamycin had a greater mitochondrial mass that was not altered by ethidium bromide (Figure 4).

**Figure 5:**
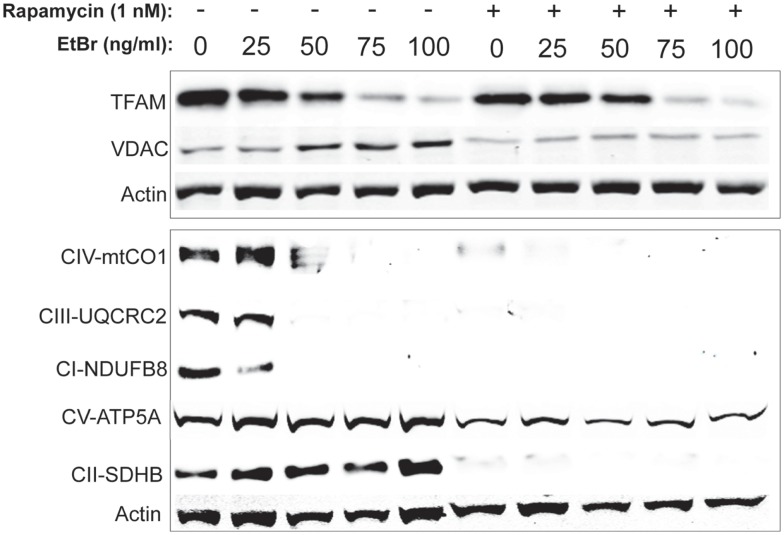
**Steady state levels of mitochondrial proteins following ethidium bromide exposure**. Human fibroblast cell cultures grown in the presence or absence of 1 nM rapamycin were exposed to increasing doses of ethidium bromide for a period of 7 days. At this time, total cellular protein extracts were prepared and analyzed by immunoblot as described in Section “Materials and Methods” for a subset of mitochondrial resident proteins. The levels of beta-actin are presented as a control for equal protein loading. The immunoblot presented is a representative blot of a minimum of two measurements with similar results.

In order to gain further insight into the changes in mitochondrial mass following ethidium bromide exposure, we examined the steady state levels of several resident mitochondrial proteins. Consistent with the reduction in mitochondrial DNA content, the steady state levels of the mitochondrial transcription factor (TFAM) decreased in both control and rapamycin treated HDFs upon exposure to ethidium bromide (Figure 5). In contrast, the steady state levels of the VDAC increased in control HDF cultures upon exposure to ethidium bromide (Figure 5). The baseline levels of VDAC were lower than controls in rapamycin treated cultures but these levels did not change upon exposure to ethidium bromide (Figure 5).

We next examined the steady state levels of a subset of the ETC proteins. The NADH dehydrogenase (ubiquinone) 1 beta subcomplex subunit 8 (NDUFB8) of complex 1, ubiquinol–cytochrome *c* reductase core protein II (UQCRC2) of complex III, and cytochrome *c* oxidase subunit I (mtCO1) of complex IV were all reduced following exposure to ethidium bromide (Figure 5). In contrast, the steady state levels of succinate dehydrogenase (ubiquinone) iron–sulfur subunit (SDHB) of complex II and the ATP synthase alpha subunit 1 (ATP5A), were not affected by exposure to ethidium bromide (Figure 5). In HDF cells treated with rapamycin, the steady state levels of all ETC proteins was lower than control cultures under normal growth conditions except for the levels of ATP5A, which were similar to controls and were not affected by ethidium bromide (Figure 5).

**Figure 6:**
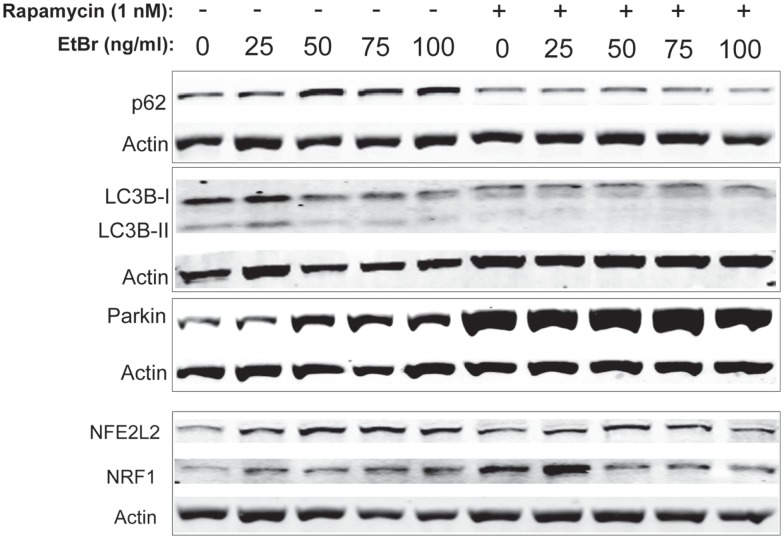
**Steady state levels of proteins involved in mitochondrial clearance and biogenesis following ethidium bromide exposure**. Human fibroblast cell cultures grown in the presence or absence of 1 nM rapamycin were exposed to increasing doses of ethidium bromide for a period of 7 days. At this time, total cellular protein extracts were prepared and analyzed by immunoblot as described in Section “Materials and Methods” for a subset of mitochondrial resident proteins. The levels of beta-actin or beta tubulin are presented as a control for equal protein loading. The immunoblot presented is a representative blot of a minimum of two measurements with similar results.

We examined proteins associated with autophagy in HDF cultures exposed to ethidium bromide. The steady state level of p62 sequestasome 1 (p62/SQSMTM1) increased in HDF cultures exposed to ethidium bromide but was uniformly lower in HDF cultures that had been treated with rapamycin (Figure 6). Both the unconjugated form of microtubule-associated protein light chain 3(LC3B-I) and the conjugated form of LC3 (LC3B-II) decreased following exposure to ethidium bromide (Figure 6), while rapamycin treated cultures showed reduced levels of both LC3B-I and LC3B-II that were not affected by exposure to ethidium bromide. We also examined the steady state levels of Parkin, a protein involved in mitochondrial turnover (30–32). The steady state levels of Parkin increased following exposure to ethidium bromide, while Parkin levels were uniformly elevated in rapamycin treated cultures relative to control cultures (Figure 6). The levels of both NRF1 and NFE2L2 (NRF2) were increased following exposure to ethidium bromide. Rapamycin treatment increased the levels of both proteins in normal growth conditions, consistent with our previous observations (29), while ethidium bromide exposure did not increase the levels of NRF1 and NFE2L2 further (Figure 6).

**Figure 7:**
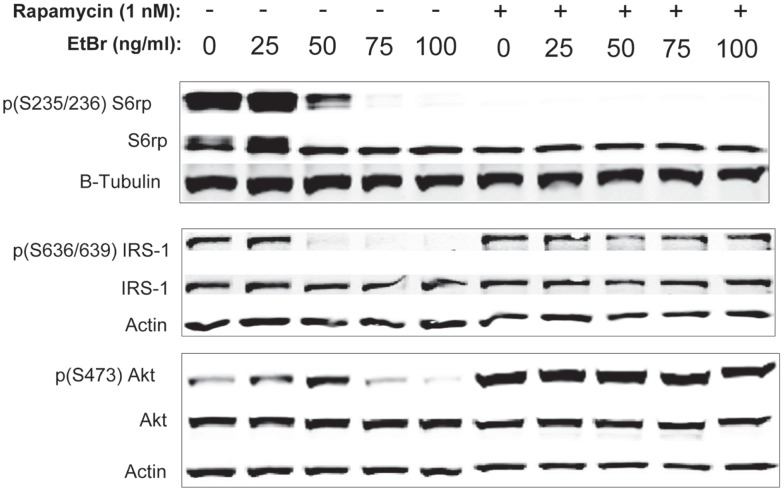
**Phosphorylation state of ribosomal S6, IRS-1, and Akt proteins following ethidium bromide exposure**. Human fibroblast cell cultures grown in the presence or absence of 1 nM rapamycin were exposed to increasing doses of ethidium bromide for a period of 7 days. At this time, total cellular protein extracts were prepared and analyzed by immunoblot as described in Section “Materials and Methods” for a subset of mitochondrial resident proteins. The levels of beta-actin are presented as a control for equal protein loading. The immunoblot presented is a representative blot of a minimum of two measurements with similar results.

**Figure 8:**
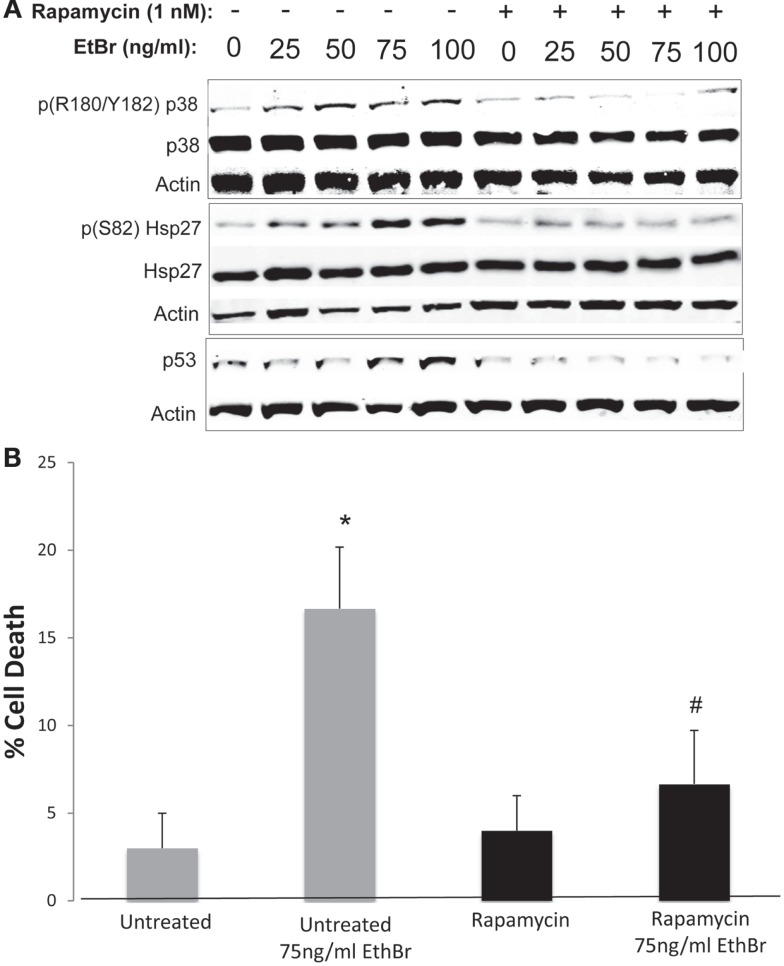
**Stress kinase signaling, p53 levels, and cell viability following ethidium bromide exposure**. Human fibroblast cell cultures grown in the presence or absence of 1 nM rapamycin were exposed to increasing doses of ethidium bromide for a period of 7 days. **(A)** Total cellular protein extracts were prepared and analyzed by immunoblot as described in Section “Materials and Methods” for a subset of mitochondrial resident proteins. The levels of beta-actin are presented as a control for equal protein loading. The immunoblot presented is a representative blot of a minimum of two measurements with similar results. **(B)** Cells were examined for viability using a trypan blue exclusion assay. Gray bars indicate control cultures and black bars indicate rapamycin treated cultures. The concentration of ethidium bromide is indicated beneath each bar. Differences between cultures exposed to ethidium bromide and unexposed cultures, which are significant at *P* < 0.05 are indicated by an asterisk and differences between control and rapamycin treated cultures under identical conditions are indicated by a pound sign.

We examined downstream targets of the mechanistic target of rapamycin (mTOR) pathway, and found the serine phosphorylation of S6 ribosomal protein, the insulin receptor substrate 1 (IRS-1), and Akt were all reduced following exposure to ethidium bromide consistent with reduced mTOR activity (Figure 7). In addition, the phosphorylation of the p38 MAPK stress kinase was increased by exposure to ethidium bromide as was the phosphorylation of the p38MAPK downstream target, heat shock protein 27 (hsp27) (Figure 8A). Levels of p53 protein were increased in cells exposed to ethidium bromide (Figure 8A) while neither the phosphorylation of 38, hsp27, nor the increase in p53 occurred if cells were treated with rapamycin (Figure 8). Treatment of cells with rapamycin also significantly increased viability at 7 days of exposure to ethidium bromide at the highest concentrations used in our studies (Figure 8B).

## Discussion

Ethidium bromide induces a cell stress that is caused by progressive loss of mitochondrial function, membrane depolarization, and ROS production. These results are counteracted by the cell in an attempt to maintain vital aspects of mitochondrial activity while targeting problematic mitochondria for clearance. Autophagy is an important pathway for the clearance of dysfunctional mitochondria and a recent report indicates that mitochondrial clearance following exposure to ethidium bromide occurs through autophagy (33) suggesting that autophagy is likely to be an important aspect of cellular responses to depletion of mitochondrial DNA.

Inhibition of the mTOR complex by treatment of cells with rapamycin is a strong inducer of autophagy and appears to improve mitochondrial homeostasis. Rapamycin treatment increases lifespan in mice, and cultured human cells, and prevents the progressive loss of mitochondrial membrane potential that normally occurs with increasing passage number (29, 34, 35). In addition, rapamycin alleviates mitochondrial disease in a mouse model of Leigh syndrome (36). Here, we find that rapamycin treatment of human fibroblasts also preserves mitochondrial membrane potential when cells are exposed to ethidium bromide and reduces both mitochondrial ROS production and total cellular ROS. Additionally, cells treated with rapamycin showed a significant reduction in stress signaling activation (as judged by p38 MAPK activity) and an increase in viability following 7 days exposure to ethidium bromide. This suggests that some aspects of changes induced by mTOR inhibition enhanced the ability of cells to adjust to the stress of ethidium bromide. One possibility is that the relative rate of autophagy was increased in cells treated with rapamycin. We have shown that treatment with nanomolar concentrations of rapamycin enhanced autophagy (29). In contrast, the increase in steady state levels of p62/SQSTM1 in cells exposed to ethidium bromide suggests an inhibition of autophagy (see Figure 6), since p62SQS/TM1 levels generally reflects the rate of autophagy, due to the fact that this protein facilitates delivery of proteins to the autophagosome and is itself degraded during this process (37). In contrast, cells treated with rapamycin exhibited a relatively low level of p62/SQSTM1 consistent with enhanced autophagy together with an elevated level of Parkin, and it is possible that this shift in autophagy was protective to cells exposed to ethidium bromide.

A second possibility for the protective effects of rapamycin may be a more efficient flux through the ETC. Previous work from our laboratory indicates that rapamycin treatment reduced electron transport chain activity but preserved functionality in the face of oxidative challenge (29). Our current analysis of electron transport chain proteins revealed that steady state levels of several ETC components were reduced but both SDHB and ATP5A were maintained when cells were exposed to ethidium bromide (see Figure 5). In contrast, in cells that had been treated with rapamycin, the steady state levels of the majority of ETC components were reduced relative to control cells. One difference in the rapamycin treated cells at baseline (no ethidium bromide) was that the relative level or ATP5A to other ETC components was increased. It is possible that the altered stoichiometry of the ETC components differed between control and rapamycin treated cells contributed to the reduced mitochondrial and cellular ROS levels relative to control cultures (compare untreated cells in gray bars to rapamycin treated in black bars in Figure 2).

A third, not mutually exclusive, possibility underlying the protective effects of rapamycin involves the differences in phosphorylation status of key second messenger proteins. Exposure to ethidium bromide caused a decrease in the phosphorylated form of the ribosomal S6 protein, suggesting an inhibition of mTORC1 activity. In addition, exposure to ethidium bromide caused a reduction in serine phosphorylation of both IRS-1 and Akt1 while the phosphorylation status of these proteins was maintained in cultures treated with rapamycin (Figure 7). Inhibition of mTOR has been shown to inhibit proteasomal degradation of IRS-1 (38–40) enhancing signaling through the PI-3 kinase/Akt pathway. Because Akt is known to antagonize apoptotic processes and has a complex relationship with mTOR signaling as it is both regulated by TORC2 and acts to regulate TORC1 activity (41), differences in Akt activity could contribute to the enhanced survival of cells treated with rapamycin. The increase in Akt phosphorylation is interesting when considered in the light of the reduced growth rate of cells maintained in the presence of rapamycin (29). In previous work, we have reported that proliferation of the human fibroblasts was significantly reduced under our culture conditions (full growth medium plus 1 nM rapamycin) but the culture was able to attain a higher number of population doublings prior to entering the senescent state (25, 29). In addition, rapamycin treated cultures displayed an increase in mitochondrial biogenesis and a reduction in the mitochondrial membrane depolarization that we and others have observed at higher population doublings in human fibroblasts (29, 42). In the present study, cultures were exposed to ethidium bromide shortly after seeding and were allowed to proliferate for 7 days. Interestingly, when cells were exposed to ethidium bromide with and without rapamycin treatment, we observed that the cell number was higher in rapamycin treated cultures after 1-week NRTI treatment period. We reported a similar paradoxical increase in clonal growth of cells treated with rapamycin when cells were exposed to IGF-1 while maintained in a non-dividing state (25) prior to reseeding in full growth medium. As with the present study, several parameters of mitochondrial function were improved in these conditions suggesting that rapamycin enhanced mitochondrial homeostasis. Thus, it appears that the protective effects of rapamycin are complex, involving shifts in phosphorylation cascades, steady state levels of ETC proteins, reduced proliferation, and an increased rate of autophagy leading to greater viability.

In summary, our results indicate that ethidium bromide exposure induces a loss of mitochondrial DNA, a reduction in several ETC protein levels, and increased ROS while increasing mitochondrial mass. We interpret this increase in mitochondrial mass as reflective of a mechanism that endeavors to maintain mitochondrial activity but which cannot compensate for the progressive loss of mitochondrial-specific gene expression, resulting in a progressive loss of mitochondrial membrane potential concomitant with an increased ROS production. Treatment of cells with rapamycin created a situation in which cells were better able to adapt to the mitochondrial dysfunction, resulting in decreased ROS and increased cell viability.

## Conflict of Interest Statement

The authors declare that the research was conducted in the absence of any commercial or financial relationships that could be construed as a potential conflict of interest.

## Supplementary Material

The Supplementary Material for this article can be found online at http://www.frontiersin.org/Journal/10.3389/fendo.2014.00122/abstract
