# Supplementary material for: Inhibition of mTOR Prevents ROS Production Initiated by Ethidium Bromide-Induced Mitochondrial DNA Depletion
Source: Front Endocrinol (Lausanne). 2014 Jul 24;5:122. doi: 10.3389/fendo.2014.00122 (PMC4109433; doi:10.3389/fendo.2014.00122)

Figure S1. Mitochondrial network imagining following ethidium bromide exposure.

Human fibroblasts containing a mitochondrial expressing GFP were grown in the presence or absence of 1 nM rapamycin. Cells were exposed to 75ng/mL ethidium bromide for a period of 7 days and imaged using an EVOS inverted epi-fluorescence microscope. Panel A and B show control cells and control cells in the presence of 1nM rapamycin , respectively, both represent an intact mitochondrial network. Panel C shows cells treated with 75ng/mL ethidium bromide and Panel D shows 75ng/ml ethidium bromide treatment supplemented with 1nM rapamycin. All images shown at 60x magnification.

Figure S1

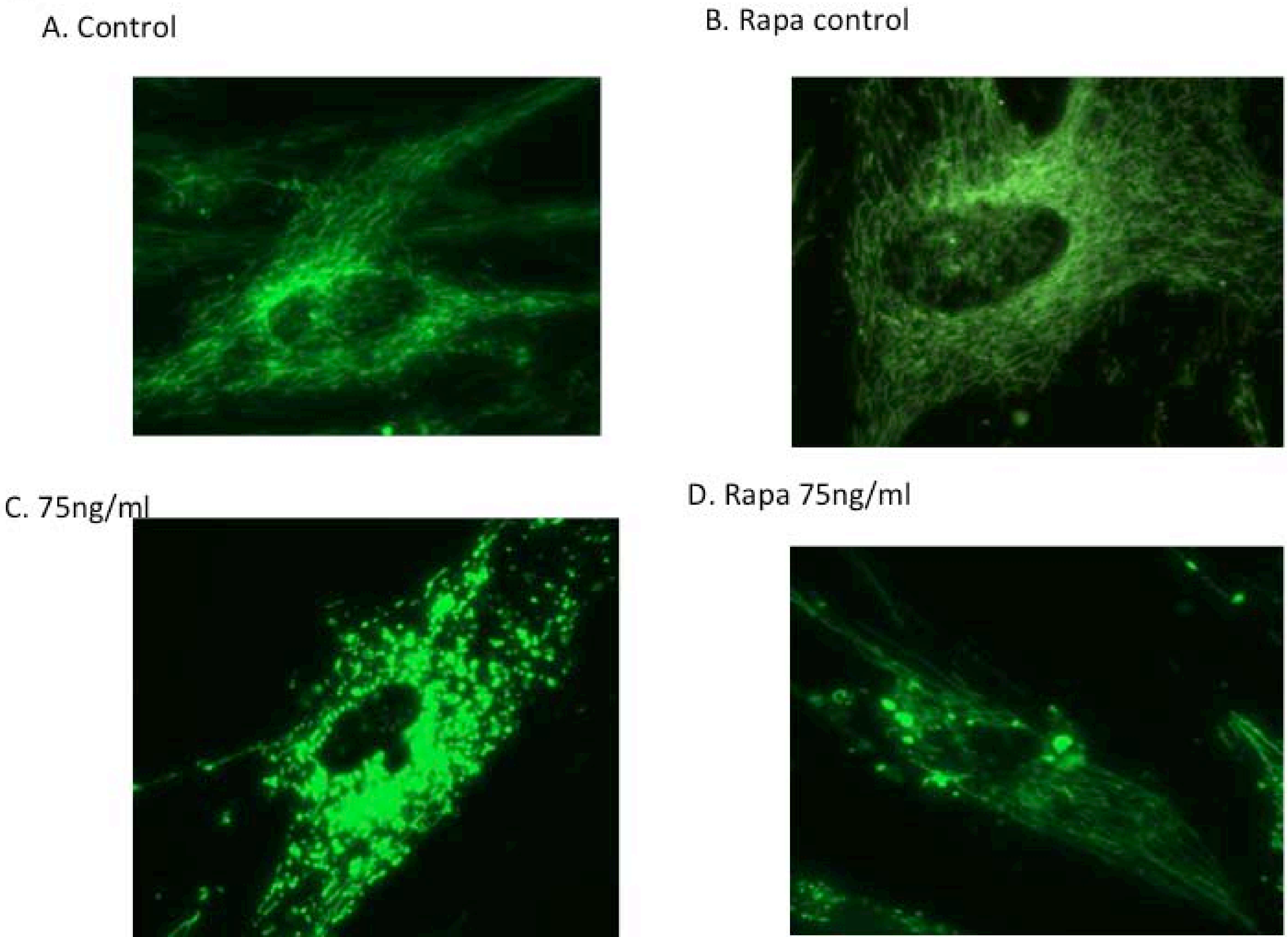

Supplement: Supplementary file 1 [file Presentation_1.PDF]
